# Quality and Findings of Bitewing Radiographs among Twenty-Year-Old Conscripts in Finland

**DOI:** 10.1155/2021/8894917

**Published:** 2021-02-08

**Authors:** Miika Hyvönen, Arttu Jaakkola, Tarja Tanner, Jari Päkkilä, Antti Kämppi, Pertti Patinen, Leo Tjäderhane, Annina Sipola, Sisko Huumonen, Vuokko Anttonen

**Affiliations:** ^1^Department of Cariology, Endodontology and Pediatric Dentistry, Research Unit of Oral Health Sciences, University of Oulu, Oulu, Finland; ^2^Medical Research Center, University of Oulu and Oulu University Hospital, Oulu, Finland; ^3^Department of Mathematical Sciences, University of Oulu, Oulu, Finland; ^4^Department of Oral and Maxillofacial Diseases, University of Helsinki, Helsinki, Finland; ^5^Centre for Military Medicine, Finnish Defense Forces, Riihimäki, Finland; ^6^HUS, Helsinki University Hospital, Helsinki, Finland; ^7^Research Unit of Oral Health Sciences, University of Oulu, Oulu, Finland; ^8^Department of Radiology, Oulu University Hospital, Oulu, Finland; ^9^Institute of Dentistry, University of Eastern Finland, Kuopio, Finland

## Abstract

**Objectives:**

Bitewing radiographs are mainly used to confirm clinical findings in caries diagnostics. The objective here was to investigate the quality of bitewing radiographs after short brush-up training and additional findings besides caries in a low-caries population.

**Methods:**

The material of this cross-sectional study comprised 377 pairs of bitewing radiographs of 19- to 20-year-olds taken by dentists. Radiography was considered indicated if one dentinal caries lesion was present on clinical examination. A senior oral radiologist evaluated quality and diagnosed the findings afterwards unaware of clinical status. The association between variables was analysed using cross tabulation and chi-squared testing.

**Results:**

Almost half of the images were of compromised quality (44.1%). Dentinal caries lesions were detected in 82.3% and enamel lesions in 73.5% of the subjects. On average, the subjects had 1.7 (SD 0.52) dentinal lesions. Fillings were found in 81.8%, fractures/cracks in 11.7%, and attrition in 7.4% of the subjects. Signs of excessive bite force were recorded in 19.4%, whereas marginal bone loss was detected in 6.4%. No significant correlation was detected between fractures, attrition, and excessive bite forces.

**Conclusions:**

Effort must be taken to ensure high quality of bitewing radiographs. In addition to caries detection, bitewing radiographs offer additional value, such as detecting excessive bite forces, tooth wear, and marginal bone loss among young adults.

## 1. Introduction

Radiographic imaging is mainly used to examine hard tissue structures. It is an inexpensive and easy-to-use method that is commonly employed in everyday dental practice to support clinical findings. As excessive radiation exposure is hazardous to health, there must always be a clinical indication for the exposure and all information from the radiographs should therefore be utilized. Besides data on caries lesions of different depths, bitewing radiographs may also offer more information, e.g., in the state of alveolar bone and other bony structures.

Bitewing radiography is a method of imaging where premolar and molar crowns and alveolar bone are exposed separately on both sides. Radiographic imaging is accomplished by placing a receptor inside and parallel to the dental arch next to the desired area, usually by using a specific holder which helps positioning of the X-ray tube [1]. The effective dose received during bitewing radiography ranges between 1–10 *μ*Sv/exposure, which is roughly equivalent to one to two days' background radiation [2, 3]. The calculated effective dose is dependent on factors such as beam size, collimator, energy, and the tissue weighting factors used (information confirmed by the Finnish Radiation and Nuclear Safety Authority STUK 2 April 2014).

The most common indication for bitewing radiography is dental caries [4]. Radiographic imaging improves especially the detection of approximal lesions [4, 5], but also lesions in occlusal surfaces, specifically among adolescents [4, 6]. The Finnish Current Care Guidelines for controlling dental caries (2020) [7] define radiographic imaging as justified in children when even one caries lesion penetrating into dentin is found in visual clinical examination. Radiographic imaging is also justifiable if there is a reason to suspect that the patient has an elevated risk of dental caries and radiographic images have not been taken in years. In countries with low-caries prevalence, radiographic imaging should never be used for screening patients [4].

The value of bitewing radiography in detecting interproximal caries lesions has been considered indisputable. For instance, Poorterman et al. (2000) [5] reported that bitewing radiography doubled the number of lesions detected. Hopcraft and Morgan [8] reported that while 22.9–32.9% of approximal caries and 75.9–82.9% of occlusal caries are detected by clinical examination, as much as 93.1–97.1% of approximal caries and 33.1–42.6% of occlusal caries are detected by radiographic examination alone. With the decreasing prevalence of dental caries in the industrialized world, the benefit of bitewing radiography is also changing. As caries lesions develop more slowly, the prevalence of clinically undetected caries is assumed to increase [9]. In deciduous teeth, the additional benefit of bitewing radiography in caries diagnostics has been reported being 27%. Periodontal disease, shown by alveolar bone loss, can also be diagnosed relatively early on bitewing radiographs [10]. In addition to caries lesions and alveolar bone loss, there is only vague, if any, evidence in literature on other findings of bitewing radiographs.

Radiography is prone to many technical errors. During the examination, the problems encountered include unsatisfactory projection due to the misdirection of the beam, incorrect receptor placement, under- or overexposure, and patient movement [11]. In conventional film radiography, the developing process is delicate and also a possible source of poor image quality. Common causes of problems with conventional films are processing errors such as under- or overdevelopment caused by inaccurate timing or temperature, problems with the developing apparatus or the solutions used (ageing, contamination), inadequate development environment, improper storing of films, or mishandling of films [11]. Many of the problems in developing conventional radiographs are avoided by using digital radiography, and the developing time is also reduced so that images are available almost immediately. The diagnostic value of both methods is similar [1, 12], but the area covered may be smaller in digital imaging using fully digital sensor plates [1].

This study aimed to reveal what additional diagnostic benefits in addition to caries lesion detection could be achieved from bitewing radiographs in regard to the periodontal state, the signs of bruxism, chipping, attrition, and root fillings. Another objective was to investigate the technical quality of the radiographs. The hypothesis was that more than one caries lesion would be detected on the bitewing radiographs in all subjects when the indication for imaging was at least one visually detected dentinal lesion in the dentition. Another hypothesis was that approximately 10% of the examined patients would have changes in the height of alveolar bone indicating periodontal disease, and additionally, signs of the strong occlusal forces and fractures in fillings would be detected.

## 2. Material and Methods

The material in this study is based on a collaboration project between the University of Oulu and the Finnish Defence Forces named “The Oral Health of the Conscripts 2011.” A population-based cross-sectional study was carried out in January and July 2011. It included an examination of conscripts' oral health as part of the obligatory general health inspection during the first weeks of military service. The total number of conscripts examined was 13,819: 13,564 men and 255 women. Twenty of the 24 Finnish military garrisons took part in this study. In the five largest garrisons, every fifth conscript in alphabetical order was examined. In smaller garrisons, all draftees were examined. Oral health examinations were carried out by fifteen dentists: twelve working in the Defense Forces, one dentist conscript doing his military service, and two research dentists from the University of Oulu [13].

BW radiographs were taken during the oral health screening of every fifth conscript with clinical indication for radiography or with at least one clinically detected active dentinal caries lesion as recommended in the Finnish Current Care Guidelines in Controlling Dental Caries [7]. Eleven garrisons had suitable equipment for intraoral radiography. There was a limitation on examination time use, so every fifth examined subject meeting the criteria was radiographed. This selection was done in alphabetical order, creating a random sample. Dentists carrying out the clinical and radiographic examinations were trained and calibrated by a senior radiologist prior to this study. On the same occasion, the protocol for radiographic practices during oral health screening was agreed upon by all participants. Instructions were available to researchers on the University's website during the research.

The mean age of the subjects was 19.7 years. The examiners had been advised to restrict the exposure area to teeth from the first premolar to the second molar. Film radiographs from all but one garrison were delivered to the University of Oulu to be analysed and developed at the Department of Cariological Dentistry. In one garrison, a digital system was used for 33 subjects. All conventional film radiographs were digitized using a sheet scanner in the photographic laboratory in the University of Oulu with a resolution of 1200 dpi.

Conventional number 2 oral films (Kodak Insight Dental Film®, Eastman Kodak Co., Rochester, NY, USA) and Minray DC SL-9® X-rays (Compare Networks Inc., San Francisco, CA, USA) were used in radiographing during the study. The exposure time was 0.28 sec. The examiners used standard bitewing projection holders to aid in projection. Automatised developing apparatus (Dürr Dental Periomat Plus®, Dürr Dental AG, Bietigheim-Bissingen, Germany) and developing solution (Periomat Intra, Dürr Dental AG) were used for development of radiographs. Digital plates were scanned by using a radiography plate scanner (Scanora system, Hammasväline, Helsinki, Finland).

A senior specialist in oral and maxillofacial radiology analysed the radiographs in the University of Oulu by using Irfanview4.32 (Wiener Neustadt, Austria) software and a monitor calibrated for analysing radiographs. Technical quality was evaluated considering the placement of the detector, the vertical and horizontal projection of the tube head and how well the desired area was seen in the image (adequate/not adequate), exposure time (adequate/not adequate), and the quality of the developing process (good/poor). The data were considered missing if only part of the tooth was seen on the image and the tooth thus had no diagnostic value. The parameters evaluated from each tooth separately were dental caries in enamel, dentin or root structure, restorations (tooth-colored restorative, amalgam, or prosthetic crown), root fillings, fractures/chipping, excess material and overhangs, microleakage of fillings, and resorption (internal and external). The radiographed area as a whole was also evaluated for the loss of alveolar bone, amount of tooth wear, changes in lamina dura with the dilation of periodontal ligament space, and the amount of dental calculus. The criteria for registering all findings are described in detail in Table 1.

### 2.1. Statistical Analysis

The data were described as frequencies and proportions of the subjects with specific findings. Mean (SD) of dentin caries lesions was calculated. Proportions and distributions of caries lesions, restorations, and endodontic treatments were calculated and illustrated using bar plots. Distribution of tooth wear, fractures/chipping, marginal bone loss, and visible calculus were also analysed. Cross tabulation with the chi-squared test was used to analyse the association between the existence of attrition, lamina dura, and fractures. Difference between the groups was considered statistically significant with *p* values lower than 0.05. A Lorenz curve was drawn to illustrate caries polarization among subjects. Statistical analyses were executed using SPSS predictive analytics software (version 20.0, SPSS Inc., Chicago, IL, USA) and R software (version 2.15, a language and environment for statistical computing; R Foundation for Statistical Computing, Vienna, Austria, URL: http://www.R-project.org).

### 2.2. Ethical Considerations

The study protocol has the approval of the Regional Ethics Committee of Northern Ostrobothnia Hospital District (dated 22^nd^ March 2013, complemented on 29 March 2013). Research permission was granted by the Center for Military Medicine on 23 June 2013. A cooperation research agreement between the Center for Military Medicine and the University of Oulu was signed on 4 April 2011. For the analyses, the personal identifiers, IDs, were excluded. The draftees gave permission to use the information about their patient documents by responding to the computer-based questionnaire.

## 3. Results

The material comprised 384 pairs of bitewing radiographs. Of these, six pairs were rejected because of poor image quality, and one due to unsatisfactory recording of the data. The final data set comprised 377 pairs of bitewing radiographs. Only 55.9% of the original 383 pairs of bitewing radiographs were of acceptable quality in terms of both projection and development of the images. Technically, 61.9% of the image pairs were of sufficient quality. As for chemical processing, 83.0% of the image pairs had been developed in an acceptable manner. Due to the errors in the projection and position of the image, some teeth, mostly the first premolars and upper second molars, were not detectable for the analyses (Table 2). No difference in quality was found between conventional film radiographs that were digitized later and those taken using a fully digital system. There was no information on whether repeated imaging exposure had taken place due to initially failed imaging.

**Figure 1:**
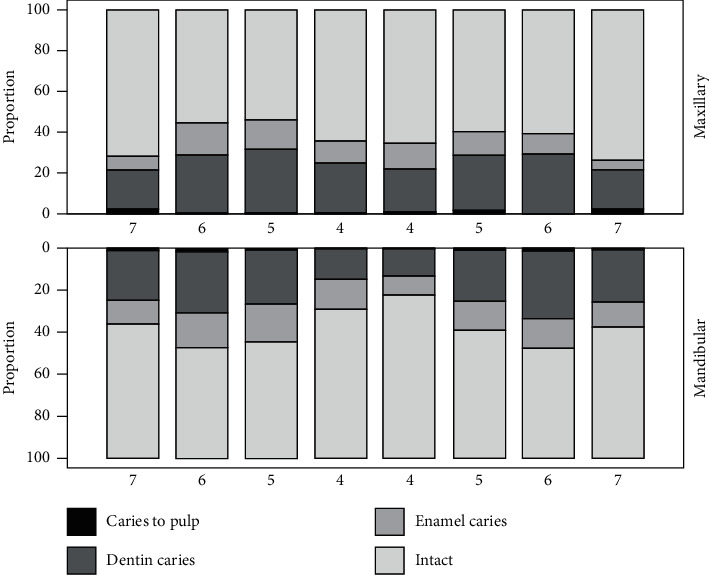
Distribution of caries lesions of different depth in maxillary and mandibular premolar and molar teeth.

**Figure 2:**
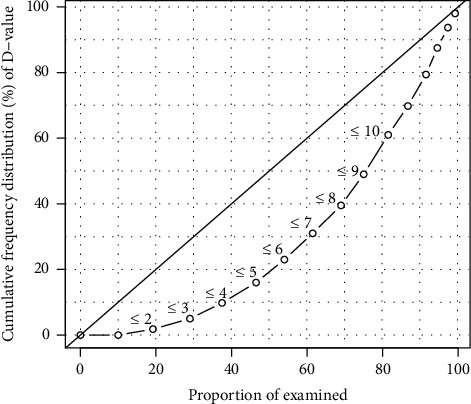
Lorenz curve illustrating distribution of the individuals with different restorative treatment need.

On average, the subjects had 1.7 (SD 0.52) dentinal caries lesions on bitewing radiographs. About 10%, or 37 subjects, had no lesions of any kind. There were 309 (82.0%) subjects with dentinal and 277 (73.5%) with enamel lesions. The majority of dentinal caries lesions were found in the lower first molars and upper right second premolars while the least amount was found in the lower first premolars (Figure 1). The worst fifth of the participants had at least ten lesions and approximately one-quarter had half of the total number of lesions. The distribution of lesions among the participants is presented as a Lorenz curve in Figure 2.

**Figure 3:**
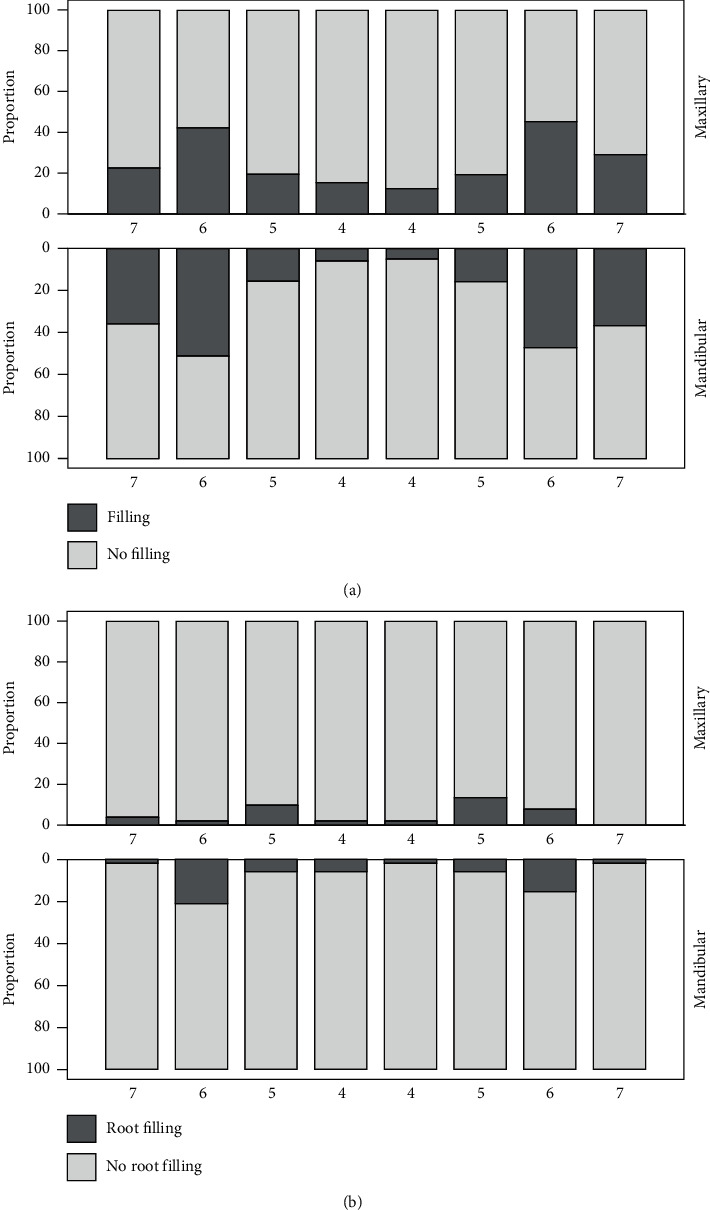
Distribution of fillings (a) and root fillings (b) in maxillary and mandibular premolars and molars.

The distribution of fillings in different teeth is presented in Figure 3(a). Fillings were present in four-fifths of the study population (81.8%). Amalgam fillings were almost nonexistent; only eight subjects had amalgam restoration. Almost forty percent (36.1%) had five or more tooth-colored restorations, while the proportions of those with one to four restorations varied only slightly from 10.6% to 11.9%. Every fourth (24.6%) participant was found to have at least one secondary caries lesion. Most restorations were detected in the first molars, the lower teeth having more restorations than the upper ones. The least amount of restorations was detected in the lower first premolars.

One in ten (10.3%) of the subjects had had endodontic treatment: 7.2% had one endodontically treated tooth while 3.2% had two or more. The absolute number of endodontically treated teeth (*n* = 52) was very in comparison with all teeth in the radiographs (*n* = 5,633). The distribution of endodontically treated teeth is presented in Figure 3(b).

The fractures/chipping of teeth were present in 11.7% of the subjects and in 8.5% of the teeth, mostly in the first molars. Almost one in ten (8.6%) had one fractured tooth; 3.1% had two or more. Tooth wear was found among 7.4% of the subjects, and in all but one case, the tooth wear was mild. Almost one in five subjects (19.4%) showed the signs of excessive occlusal stress recorded by evaluating the increased size of lamina dura and dilation of the periodontal ligament space. There was no association between the signs of tooth wear (attrition, abrasion, erosion) and occlusal stress. In addition, there was no association between the tooth chipping and signs of tooth wear or occlusal stress.

As for periodontological findings, minor alveolar bone loss was found in 6.4% of the subjects while moderate and severe bone loss was found in one subject each. Calculus was visible in 6.1% of the subjects: on one or two teeth in 3.7%, on three or four teeth in 1.1%, and on five or more in 1.3% of the subjects. No association between calculus and alveolar marginal bone loss was detected.

## 4. Discussion

Both dentinal caries lesions and restorations were present in more than 80% of the participants and more than 10% had at least one root filling. Polarization was detected among the participants, as was expected. Despite recent brush-up training, the quality of images was good in only two-thirds of the cases. Bitewing radiographs offered also other information; alveolar bone loss indicating periodontal disease was seen at least in some degree in 6.5%. Additionally, fractures and signs of strong occlusal forces and fractures in fillings were detected (9% and 19%, respectively).

The aim was to investigate the technical quality of the radiographs. The main problems encountered in the quality here were due to the misdirection of the X-ray beam or wrong exposure time. Almost 45% of the radiographs were of compromised quality. The importance of the projection is emphasized, especially when using one receptor per side compared with two. Here, the examiners used devices aiding in beam-aiming which help to achieve correct projection [1]. However, the time used for the examination was limited and may have influenced the outcome. There was no information as to how often an extra image had to be exposed due to failure in exposing the first one, which is a limitation. Extra radiographs always cause unnecessary radiation, which must be emphasized in training dentists for radiography. Indeed, noninvasive new methods should be used whenever possible.

The study revealed no difference between conventional film bitewing radiographs that were later digitized and radiographs that were taken using a fully digital system. The effect of digital radiography regarding the quality and diagnostic value of the images has been extensively studied. In 1997, White et al. showed that film and digital systems are diagnostically comparable, for instance, in detecting approximal enamel caries lesions [14]. Diagnosing radiographs is challenging and variation among the examiners exists [15]; however, that could not be studied here.

The effect of digital radiography in increasing the number of radiographs taken is a topic of debate. Digital radiography can be done either indirectly or directly. In indirect digital radiography, digital image plates are of similar size to conventional films. However, to date, the digital sensors in the direct system are bulkier and cover a smaller area, which leads to positioning errors and increased difficulty in achieving correct projection. This increases the number of retaken images and decreases the tooth surfaces available for analyses [16]. Digital radiography has been claimed to reduce the patient dose by as much as 50–80% due to shorter exposure times, but this advantage might be lost as dentists using digital appliances tend to take more radiographs [17]. In this study, digital imaging was possible only in one office at the time of the study. The largest group of teeth not detectable on the images was the first premolars followed by upper second molars. The fact that second molars were missing from the images may not have had much effect on the mean caries status of the study group as they have moderate caries expression compared with other teeth in bitewing radiographs in this age group. On the contrary, the first premolars missing from the images may worsen the status as the first premolars are on average healthier compared with other teeth in this age group.

In a low-caries prevalence population, the polarization of dental caries can be considered high, which was true here as 25% of the subjects had half of the total dentinal caries lesions. There was less polarization according to the radiographs than according to clinical findings [13], which is reasonable since bitewing radiographs were taken only from subjects with at least one clinically detected caries lesion. Our study indicates the same outcome as the conclusion of Hietala-Lenkkeri et al. (2014) reporting that subjects with clinically detected caries lesions benefited more from bitewing radiography than subjects with clinically caries-free dentition. Comparing outcomes by radiographic and clinical examinations is a topic for a future study [6].

Most caries lesions were found on the lower right first molars and upper second premolars. Upper first molars and lower second premolars were found to have the second most lesions. The lower first premolars and upper second molars were the least affected. The results are in concordance with those of Batchelor and Sheiham (2004) [18] and Hietala-Lenkkeri et al. (2014) [6]. The results are also pursuant to the eruption order of and thus the posteruptive age of permanent teeth.

Larmas et al. (1995) found that 10–25% of all permanent molar teeth were filled during the year following eruption [19] Five to eight years after eruption, the restoration rate was found to decrease [20]. The nature of this study is cross-sectional, and therefore, the timing of restorations could not be monitored. Here, the distribution of restorations followed the pattern of caries lesions.

Poorterman et al. (2000) reported that the benefit of bitewing radiography in connection with clinical examination was the biggest when it was used on 17-year-old subjects compared with younger (14-year-olds) or older (20, 23, 25–34, 35- to 54-year-olds) ones [5]. This is probable because, in this age group, the first interproximal caries lesions develop and only some of them are clinically detected. Poorterman et al. (2003) also found that, between 17 and 23 years of age, approximately a third of sound occlusal surfaces developed new dentinal caries lesions [21]. At the same time, over 70% of the existing lesions expressed progression. They concluded that, at the age of 17, occlusal surfaces are still very susceptible to dentine caries. Kidd and Pitts (1990) emphasized the role of bitewing radiography in detecting approximal initial caries lesions, which gives a chance for preventive instead of invasive caries treatment [22].

Amalgam restorations were almost nonexistent, which is explained by the fact that the use of amalgam in Finland declined rapidly after the recommendation of the Ministry of Social Affairs and Health in 1994 to reduce the use of dental amalgam. As a result, and possibly due to the public opinion, the use of amalgam declined, especially in children's restorative treatment, in Finland as well as in the other Nordic countries [23]. The study population was born in the early 1990s and thus was subject to this change in protocol.

Secondary caries lesions were found in every fourth subject. This is not surprising since several studies have pointed out the relatively short life span of composite restorations mostly used in restorative care. Palotie and Vehkalahti [24] (2002) reported that the mean age of tooth-colored fillings was 2.4 years, while the mean age of amalgam fillings was 8.9 years. The restorations are also placed in caries risk individuals, which poses a risk for the survival of the restoration [25].

Endodontic treatment had been carried out in less than 7% of the subjects. The most often endodontically treated teeth were the lower first molars followed by the upper second premolars, a finding in line with the cariological status. In this age group, there is very little additional value to be expected of BW radiographs regarding endodontic treatment.

Tooth fractures were quite common (12% of the subjects), whereas attrition was less common (7.4% of the subjects). It is interesting how the signs of excessive occlusal stress were surprisingly common; they were seen in one in five subjects. There was a nonsignificant association between the signs of tooth wear and occlusal stress. No association was found between the signs of tooth wear and fractures or between occlusal stress and fractures. We could not find any literature on the signs of occlusal stress on bitewing radiographs; it would be an interesting topic for future research.

All radiographic methods underestimate alveolar bone loss. The underestimation has been found to be 11–23% in bitewing radiographs [26]. Although intraoral and panoramic radiographs offer some advantages over bitewing radiography in diagnosing bone loss, the difference is small in premolar and molar areas [26]. Douglass et al. (1986) studied the sensitivities and specificities of the three different radiographic methods mentioned and reported somewhat similar sensitivities but a significant difference in specificity [27]. Panoramic imaging had the lowest specificity values compared with periapical and bitewing radiographs which were quite similar. Corbet et al. (2009) suggested the use of vertical bitewings instead of horizontal ones in periodontal treatment planning [28]. Here, periodontological findings were less prevalent than anticipated. An examination made in Swedish recruits [29] revealed radiographically visible alveolar bone loss in 13% of the subjects. Bäckman (1981) found bone loss at the first molars to be prevalent in 8% of the national servicemen studied [30]. Kållestal and Matsson (1991) studied bitewing radiographs for alveolar bone loss in 16-year-olds and found the signs of early periodontitis in 3.5% of the subjects [31]. Jenkins et al. (1992) concluded the alveolar bone loss in Caucasian adolescent population to be between 2% and 11%, the criterion being 2 mm or more between alveolar crest and cement-enamel-junction [32]. The findings of alveolar bone loss in the present study are in accordance with these studies. There was no association between detected calculus and bone loss, as also previously presented by Kållestal and Matsson (1991) [31].

The size of the original study population (>13,000) is quite large and representative (80% of the age cohort) of the males in this age group. Eleven garrisons out of 20 had suitable radiography equipment and due to the restrictions on examination time use, only every fifth individual meeting the criteria was radiographed. Investigating the association between clinical and radiographic findings should be a topic for future study. This study was conducted in well-equipped clinics by calibrated examiners. The young age of the participants limits the number and variety of findings. It would be interesting to have older participants in a similar study in the future or perform a follow-up study with the same study group.

## 5. Conclusions

As simple as bitewing radiography may seem for a clinician, good technical quality is challenging to achieve. Thus, continuing education must be emphasised, and regular steps should be taken towards updating radiographic skills in both how to take a successful image and how to diagnose it for full effect. Standardized protocols should also be utilized to aid in generating reproducible quality in both aspects.

For this age group and with the clinical indication as used here, bitewing radiography is an excellent tool for the early detection of caries risk patients. The clinical relevance of this study is that, in addition to cariological findings, bitewing radiography enables recognition of the first signs of periodontal disease in subjects of relatively young age. It also offers a reliable means of detecting tooth wear and tooth fractures and fillings as well as evaluating and monitoring the signs of occlusal stress: this may support early diagnosis of factors leading to temporomandibular disorders.

## Figures and Tables

**Table 1:** The variables and their values used in the study.

Pair of bitewing radiographs	
Technical quality (projection, exposure)	0 = adequate
	1 = inadequate
Chemical quality (development)	0 = adequate
	1 = inadequate

Whole dentition	
Loss of marginal bone	0 = <2 mm (healthy, normal)
	1 = 2–4 mm (minor)
	2 = 4–6 mm (moderate)
	3 = >6 mm (severe)
	4 = furcation
	5 = complicated (vertical and horizontal loss of bone)

Lamina dura (occlusal stress)	0 = normal
	1 = increased size of the lamina dura and dilation of the periodontal ligament space

Attrition (abrasion, erosion)	0 = no attrition
	1 = minor
	2 = severe

Calculus	0 = none
	1 = scarce, in one or two teeth
	2 = moderate, in two or three teeth
	3 = plenty, in five or more teeth

By teeth	
Filling	0 = no filling
	1 = tooth-colored filling
	2 = amalgam

Caries	0 = intact
	1 = enamel caries
	2 = dentinal caries
	3 = caries to pulp
	4 = root caries

Fractures/chipping	0 = no fracture
	1 = fracture

Microleakage	0 = none
	1 = leakage

Excess filling material	0 = none
	1 = excess filling

Resorption	0 = none
	1 = resorption

Endodontic treatment	0 = none
	1 = root canal filling

**Table 2:** Proportions of teeth not fully seen in the radiographs.

Tooth no.	Missing (%)
17	8.5
16	0.0
15	0.8
14	8.0
24	18.3
25	1.9
26	1.3
27	8.5
37	4.0
36	1.1
35	2.4
34	26.3
44	16.2
45	1.6
46	1.3
47	4.0

## Data Availability

Data are available for collaboration projects until December 2020 and can be required through the corresponding author. The use of data is defined by an agreement between the University of Oulu and Finnish Defence Forces.
